# WHO’s structured process for creating health-conducive environments in countries: insights and examples from the African region

**DOI:** 10.1080/16549716.2025.2596450

**Published:** 2025-12-19

**Authors:** Jennyfer Wolf, Cristina Vert-Rocca, Blanche-Philomene Melanga Anya, Abdoulaye Mariama Baissa, Bruce Gordon, Melanie Bertram, Clement Lugala Peter Lasuba, Maria Neira, Adelheid Werimo Onyango, Bernard Amon Tomas, Annette Prüss

**Affiliations:** a Department of Environment, Climate Change, Migration and One Health, World Health Organization, Geneva, Switzerland; b WHO Country Office, World Health Organization, N’Djamena, Chad; c WHO Country Office, World Health Organization, Niamey, Niger; d Department of Delivery for Impact, World Health Organization, Geneva, Switzerland; e One UN House, World Health Organization, Monrovia, Liberia; f Regional Office for Africa, World Health Organization, Brazzaville, Republic of the Congo; g Department of Country Strategy and Support, World Health Organization, Geneva, Switzerland

**Keywords:** environmental health, climate change and health, World Health Organization, implementation frameworks, cross-sectoral collaboration

## Abstract

This paper presents the structured process developed by the World Health Organization (WHO) to systematically scale up actions in environment, climate change, and health at the country level. The process is designed to implement evidence-based and data-driven actions tailored to local contexts and to bring together diverse stakeholders from various sectors such as health, environment, energy, and transport. It contains three steps: (1) analysing the country’s current situation regarding environmental exposures and associated health impacts, (2) matching priorities with effective actions integrated with ongoing activities, and (3) assisting with implementation and monitoring. Various resources support these steps, including data scorecards, checklists, and a catalogue of interventions. These also cover effective communication with stakeholders and ways for sustaining change. Country examples are presented to illustrate the practical application of this process. In conclusion, this paper highlights the critical role of upstream approaches to disease prevention and provides practical advice for advancing WHO’s goal of leaving no one behind in achieving the highest possible level of health as well as the human right to a clean, healthy, and sustainable environment.

## Paper Context

**Main findings:** The WHO’s process to enhance environment, climate change and health action in countries is presented along country examples.

**Added knowledge:** This work highlights data-driven and evidence-based cross-sectoral approaches tailored to local contexts and provides new tools and resources.

**Global health impact for policy and action:** Detailed guidance for field actors of environment, climate change and health is provided for this process.

## Background

In 2022, the United Nations (UN) General Assembly recognized a clean, healthy, and sustainable environment as a human right [1], encompassing clean air, safe water, healthy food, a safe climate, biodiversity, and non-toxic environments [2].

However, the reality contrasts this vision. Preventable environmental risks account for at least 24% of deaths [3,4]. Health systems are strained by rising costs and demand [5], exacerbated by the environmental crisis, including climate change, biodiversity loss, and pollution. Primary disease prevention through health-conducive environments is vital for tackling global health threats like non-communicable diseases and antimicrobial resistance [6].

The World Health Organization (WHO) fosters healthier environments for primary disease prevention and health promotion [7]. WHO provides technical guidance on protecting health from climate change, reducing air pollution, establishing poison centres, and implementing safe water and sanitation [8–14].

Despite extensive guidance, making these resources accessible to decision-makers and public health practitioners remains challenging [15,16]. Therefore, WHO developed a comprehensive, action-oriented process to advance environment, climate change, and health (ECH). It promotes national cross-sectoral interventions, leveraging data, guidance, standards, and coordination.

This publication presents this process and its supporting resources particularly for everyone promoting healthier environments for healthier populations.

## The process

**Figure 1.**
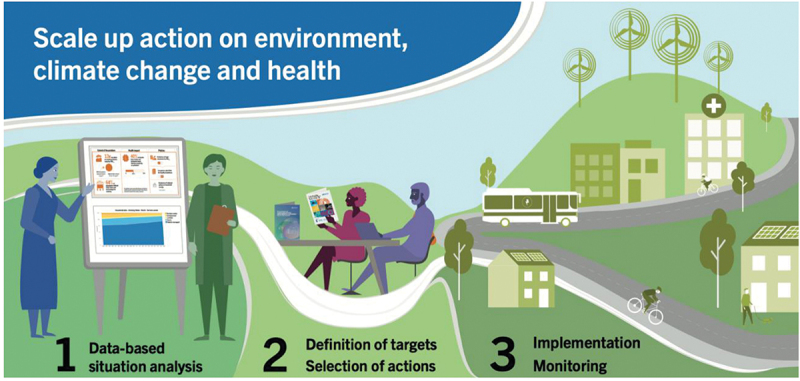
Three step framework of the WHO process for systematically scaling up environment, climate change and health action in countries. (1) analysing the country’s situation of environmental exposures, associated health impacts and policies to set data-driven priorities; (2) matching priorities with effective interventions integrated with country activities; and (3) assisting with implementation and monitoring.

**Figure 2.**
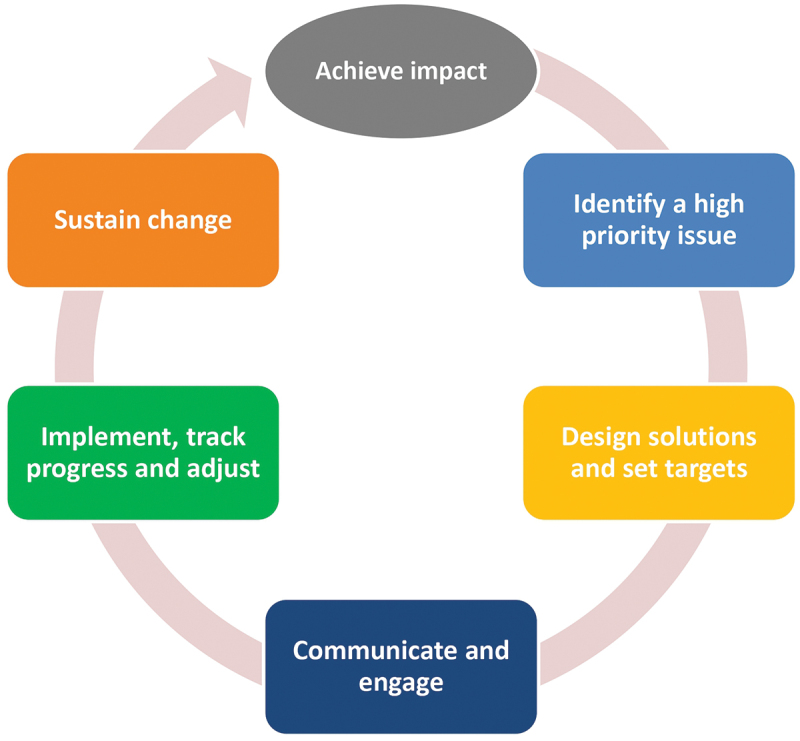
WHO’s Impact Cycle (adapted from WHO, 2024 [17]).

The process and accompanying resources aim to systematically increase country-level action in ECH, following general WHO implementation guidance [17]. It is structured in three steps (Figure 1):

These steps are based on WHO’s Impact Cycle, an iterative implementation process which uses best practices, and health systems and implementation science [17] (Figure 2).

Various resources were developed to support this process, including country-specific data scorecards [18] and checklists [19] for rapid national assessments and identifying high-priority issues (step 1 of the Impact Cycle). These can also be used for monitoring intervention implementation, tracking progress, and adjusting (step 4). A catalogue of ECH interventions [20] was developed (step 2) and is accompanied by action lists supported by WHO (Supplementary File).

To foster effective stakeholder communication, WHO developed briefings [21] covering major ECH topics and an open online course for practitioners and policymakers [22] (step 3). For sustaining change at the country-level (step 5), a webinar series covering practical action on key topics was conducted in early 2025.

## Results and country examples

Applying the process, WHO collaborated with 14 countries in the African Region (Supplementary File). Since the most effective environmental health actions are implemented in sectors beyond health, cross-sectoral national workshops were organized to bring together decision-makers from areas like health, environment, energy, industry, land use, agriculture, and transport. A key outcome was a collaborative, data-driven implementation and action plan listing key areas and activities, resources, stakeholders, timelines, and funding sources (Annex 1 of [17]).

Two country examples are presented:

In Niger, a low-income, landlocked country, a multisectoral workshop was organized involving various stakeholders and ministries, such as The National Environment Council for Sustainable Development (CNEDD) at the prime Minister’s office, and The National Institute of Agronomic Research of Niger (INRAN), Ministries of Health, Water and Sanitation, Environment, Agriculture, Mining, Transport, and Energy, along with universities, research institutes, NGOs, and representatives from WHO and other UN organizations. The environmental health situation was assessed using the health and environment country scorecard [23] and put into perspective with ongoing country activities. This highlighted major health impacts from high levels of ambient and household air pollution and poor sanitation, outlined possible solutions, selected initial cross-sectoral actions and raised awareness. Subsequently, fundraising proposals were developed on air quality monitoring and electrification of healthcare facilities. Public awareness materials were prepared on the health effects of air pollution, and an energy and electrification assessment of healthcare facilities was initiated. Energy needs assessments of health care facilities are important for planning and implementing sustainable energy solutions in the health sector, particularly for electrifying health care facilities. Unfortunately, the political situation in Niger deteriorated in 2023, delaying several planned activities. Despite this challenging context, the energy needs assessment for high-priority facilities is expected to be finalized in few months. Furthermore, the assessment in Niger helped catalyse similar work in Chad through knowledge transfer among countries.

Also in Liberia, a multi-stakeholder discussion between WHO, Ministries of Health, Labour, Mines and Energy, Justice, and local stakeholders was organized. After the initial situation analysis using country-specific data [24], focus areas were defined, actions were prioritized, and a work plan was developed encompassing an air pollution source apportionment study, the development of water, sanitation, and health standards, the establishment of a poison centre, and an assessment of climate change vulnerability and adaptation for the health system. Fundraising proposals were developed for the electrification of healthcare facilities and the implementation of the WHO Clean Household Energy Solutions Toolkit (CHEST). Guidance was provided on the ratification of the Minamata Convention on Mercury, which entered into force at the end of 2024, developing a national chemical roadmap by the Ministry of Health, and conducting a vulnerability and adaptation assessment of the health system, a national adaptation target in Liberia’s Third Nationally Determined Contribution (NDC 3.0) [25].

## Discussion and conclusions

This process advocates for increased ECH actions in countries to create health-conducive environments. It presents concrete steps towards WHO’s goal of ensuring everyone attains the highest possible level of health and the human right to a clean, healthy, and sustainable environment [2]. Based on implementation science, it advocates for evidence-based, data-driven actions tailored to the national context. The setup of the process and the supporting resources will likely benefit anyone working to improve environmental health.

Effective ECH action requires cross-sectoral collaboration, a key aim of this process. Interventions outside traditional healthcare, such as implementing renewable energies, protecting ecosystems, reducing plastics and waste, promoting sustainable healthy diets, and encouraging active modes of transport, can hugely impact population health [20,26]. Creating health-conducive environments through upstream approaches to disease prevention has proven more effective than relying on individual behaviour change [27,28]. Scaling up just five climate-health actions – heat-health warning systems, electrification of healthcare facilities, WASH for climate change adaptation, cleaner household energy sources, and fiscal policies to efficiently price fossil fuels – could save nearly 2 million lives annually and generate a return of over four US dollars for every dollar spent [29,30]. Case studies presented in this paper are two low-income countries. They face different environmental health challenges compared to wealthier countries; the structured process for advocacy as described here remains largely the same.

However, governance mechanisms that facilitate cross-sectoral collaboration are often lacking. Ministries and other decision-makers frequently operate in silos when addressing environmental health challenges. Tackling highly intersectoral ECH issues requires a distinct approach compared to more traditional health care issues. Additional barriers to national action include limited financial and human resources, short political cycles, political instability, insufficient training of health care providers and other stakeholders, and a lack of public awareness regarding environmental health risks. National health systems often demonstrate weak governance structures [16], which hinder their ability to effectively implement WHO guidance. Barriers at the level of the WHO include lacking feedback mechanisms for end-users, integrated and action-oriented guidance, and advice on implementation strategies, resource requirements, and monitoring frameworks [15,16].

At the country level, the following elements were judged essential for stimulating ECH action:

1. Strong political commitment, leadership, and support towards environmental health including a strong health system.
2. A governance structure, institutional space, or other mechanisms for cross-sectoral collaboration.
3. Sustainable funding mechanisms, such as earmarked taxes on polluting fuels and products or abolishing harmful subsidies.
4. Mechanisms to reduce financial and other undue interests, including legal and regulatory frameworks, independent oversight, and public participation.

In conclusion, this paper offers a practical approach to advocating for enhanced national action in ECH.

## Supplementary Material

Supplementary_file.docx

## Data Availability

No data were created or analysed in this study.
