# Supplementary material for: WHO’s structured process for creating health-conducive environments in countries: insights and examples from the African region
Source: Glob Health Action. 2025 Dec 19;18(1):2596450. doi: 10.1080/16549716.2025.2596450 (PMC12720683; doi:10.1080/16549716.2025.2596450)
Supplement: Supplementary_file.docx [file ZGHA_A_2596450_SM1059.docx]

# **Supplement** **to:** WHO’s structured process for creating health-conducive environments in countries: insights and examples from the African Region


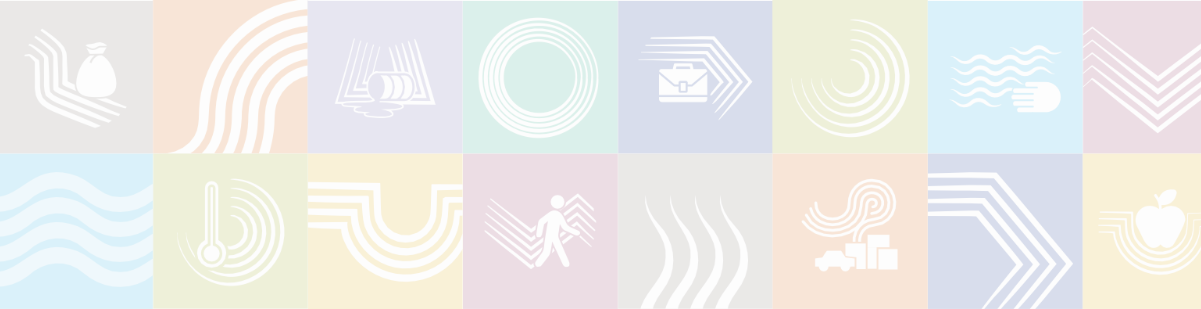

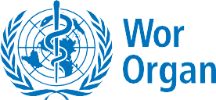

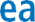

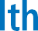

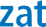

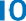

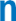


**WHO Packages of services –**

**Environment, climate change and health**

e. Foster implementation of the International Health Regulations (IHR).

| 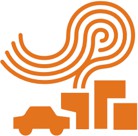 | 1. **Ambient air pollution**    1. Support air quality monitoring from the health poin access to air quality data.    2. Review regulations relating to Air Quality Guidelines    3. Manage air pollution levels through intersectoral co       - Review policies in other sectors.       - Develop a platform/support governance m intersectoral cooperation.       - Develop a strategy for air quality and prom cooperation with other sectors. | **Priority**  t of view; ensure public ☐  .  operation ☐  echanisms for ☐  ☐  ote priority actions in  ☐ |
| --- | --- | --- |
| 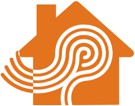 | 1. **Household air pollution**    1. Implement the Clean Household Energy Solutions develop policy action plans for expanding clean ho | **Priority**  Toolkit (CHEST) to ☐  usehold energy access |
| 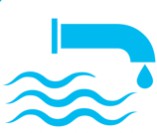 | and use.   1. **Water, sanitation and hygiene**   *Water*   - 1. Review monitoring, regulations, and standards.   2. Protect drinking-water supplies using Water Safety   *Recreational water*   - 1. Review monitoring, standards and targets.   2. Develop WSPs for priority bathing sites and pool sa   *Sanitation*   - 1. Review monitoring, regulations and standards.   2. Develop Sanitation Safety Planning. | **Priority**  ☐  Plans (WSPs). ☐  ☐  fety plans. ☐  ☐  ☐ |
| 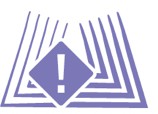 | 1. **Chemical safety**    1. Foster implementation of the WHO Chemical’s Road    2. Foster implementation of the International Health | **Priority**  Map. ☐  Regulations (IHR). ☐ |
| 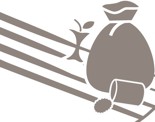 | 1. **Waste management**    1. Develop or update the solid waste management sy and regulations; Implement international agreemen    2. Promote awareness campaigns on waste reduction | **Priority**  stem; Review policies ☐  ts.  and hazardous waste. ☐ |
| 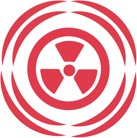 | 1. **Radiation**    1. Develop or update national sun protection policies    2. Review policies and standards that limit electromag    3. Develop or update a national radon action plan.    4. Develop or update national policies and plans for pr preparedness, monitoring, response and recovery emergencies. | **Priority**  and action plans. ☐  netic fields. ☐  ☐  evention,  after radiation ☐  ☐ |


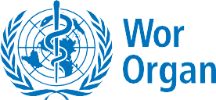

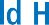

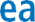

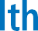

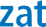

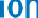


occupational health and safety for health workers at national, sub-national and facility levels, in concert with programmes for health workforce strategies and quality and safety of care.

| 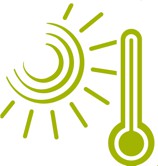 | 1. **Climate change**    1. Assess the health risks of climate change, the health co-benefits of climate action, and GHG emissions of health systems and facilities.    2. Develop plans and strategies to address climate-sensitive health risks, including Health National Adaptation Plans (HNAPs), Healthy Nationally Determined Contributions (NDCs) and Long-term Low Emissions Development Strategies (LT-LEDS).    3. Access the Green Climate Fund (GCF) Readiness Programme, and other climate finance streams, for health.    4. Implement climate change and health interventions, including:       - Climate-informed health surveillance and early warning systems.       - Climate-resilient and environmentally sustainable health systems and facilities, including assessment of climate change vulnerability, assessment of GHG emissions and environmental sustainability, development of improvement plans.       - Climate-resilient water and sanitation safety plans.       - Climate change and health capacity building.    5. Provide national evidence summaries and monitor progress through WHO/UNFCCC global survey and country profiles. | **Priority**  ☐  ☐  ☐  ☐  ☐  ☐  ☐  ☐ |
| --- | --- | --- |
| 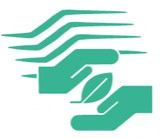 | 1. **Nature and health**    1. Assess the health risks of biodiversity loss and ecosystem degradation on human health, the health co-benefits of the sustainable, management, use and restoration of biodiversity.    2. Share and exchange best practices on health co-benefits of nature-based solutions.    3. Manage biodiversity loss impacts on human health through multi- stakeholder and intersectoral cooperation.    4. Develop and implement biodiversity and health interventions.    5. Develop the health component of National Biodiversity Strategies And Action Plans (NBSAPs) and National Reports.    6. Foster the implementation of the quadripartite alliance for One Health’s Joint Plan of Action on One Health and its associated plan for implementation. | **Priority**  ☐  ☐  ☐  ☐  ☐  ☐ |
| 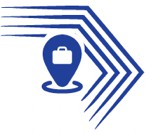 | 1. **Healthy and safe workplaces**    1. Promote national and sectoral initiatives for healthy, safe, and resilient workplaces addressing determinants of health at the workplace.    2. Foster cooperation between the health and labour sectors for healthy and safe working conditions.    3. Support expanding the coverage of workers with occupational health services as part of integrated primary health care and universal health   coverage. | **Priority**  ☐  ☐  ☐ |
| 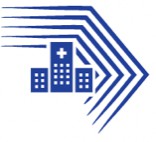 | 1. **Health care facilities**    1. Support the provision of adequate and reliable water, sanitation and hygiene services, waste management and electricity supply.    2. Support the development and implementation of programmes for | **Priority**  ☐  ☐ |


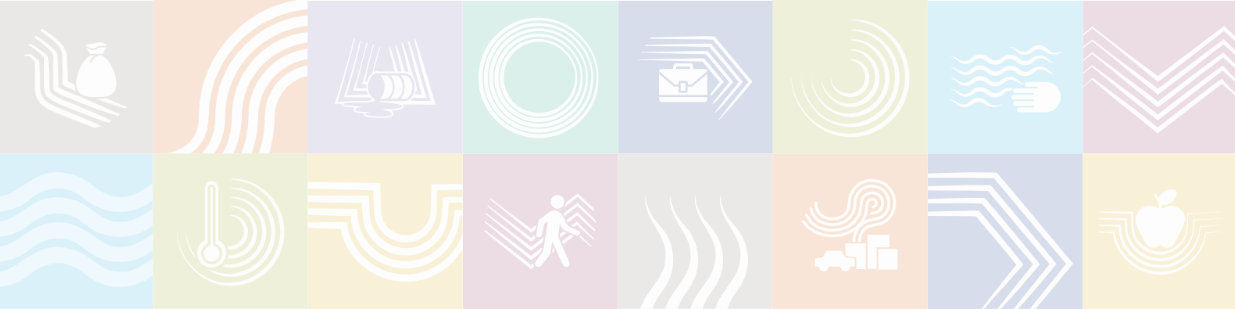

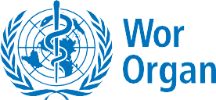

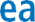

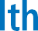

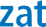

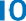

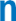


**List of actions with rapid results –**

**Environment, climate change and health**

April 2023

| 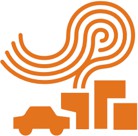 | - **Ambient air pollution**   - Identification of air pollution levels and main sources of pollution. |
| --- | --- |
| 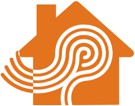 | - **Household air pollution**   - First step in implementation of CHEST tool for cleaner energy solutions for cooking. |
| 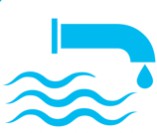 | - **Water, sanitation, and hygiene (WASH)**   - Development of the WASH accounts (WASH funding and financing).   - Translate/adapt recommendations from WHO Guidelines on Sanitation and Health into national definitions, plans and standards   - Facilitation/leadership of a national coordination group on WASH in HCF and implementation of WASH FIT |
| 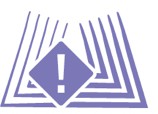 | - **Chemical safety**   - Development of a poison centre.   - Development of legislation on leaded paints.   - Development of legislation on mercury-containing skin-lightening products. |
| 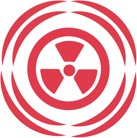 | - **Radiation**   - Development of legislation on electromagnetic fields. |
| 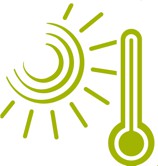 | - **Climate change**   - Development of the health component of a national adaptation plan.   - Support for joining the commitments for climate resilient for low carbon and sustainable health systems and the ATACH (Alliance for Transformative Action on Climate and Health). |
| 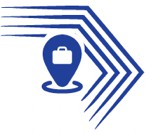 | - **Healthy and safe workplaces**   - Development of policies to protect the health and safety of health care workers. |


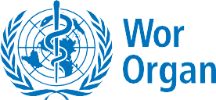

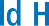

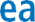

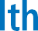

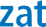

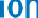


| 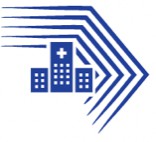 | - **Health care facilities**   - Development of an energy needs assessment.   - Implementation of WASH-FIT for water and sanitary hygiene. |
| --- | --- |

## List of 14 countries in the African Region

Guinea, Kenya, Liberia, Madagascar, Mali, Mauritania, Mauritius, Mozambique, Niger, Rwanda, Senegal, Seychelles, Sierra Leone, Zimbabwe

## List of resources supporting systematic scaling up action in environment, climate change and health

| **Health** **and** **environment** **country** **scorecards**^1^  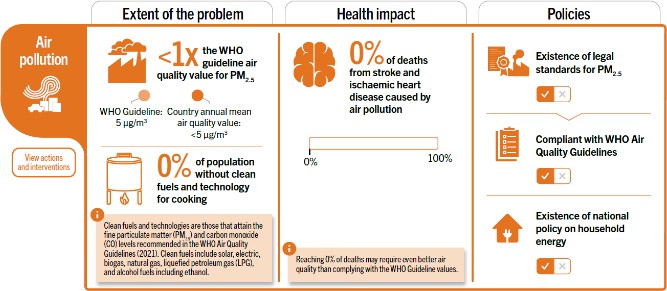 | Illustrated summary of key WHO and other United Nations (UN) data on exposures, associated health impacts and the policy situation in every country. They provide an overview of the country’s situation in key areas, including air pollution, WASH, climate change, biodiversity, chemicals, radiation, occupational health and environmental aspects of health care facilities. |
| --- | --- |
| **Checklist** **for** **rapid** **assessment** **of** **policies** **and** **actions** **in** **environment,** **climate** **change** **and** **health**^2^  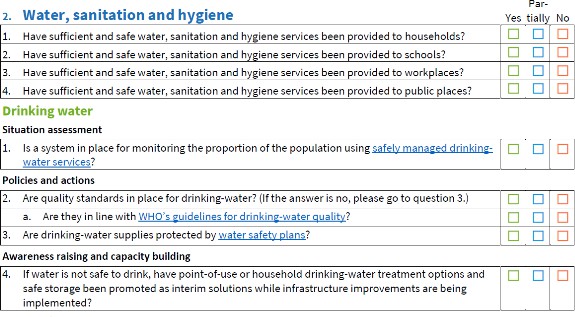 | Rapid assessment questionnaire by environmental health area which collects data of the country situation, policies, interventions, awareness raising and capacity building in place. It can be used to identify country-specific needs and gaps for action towards healthier environments. |

| **Compendium** **of** **WHO** **and** **other** **UN** **guidance** **on** **health** **and** **environment**^3^ 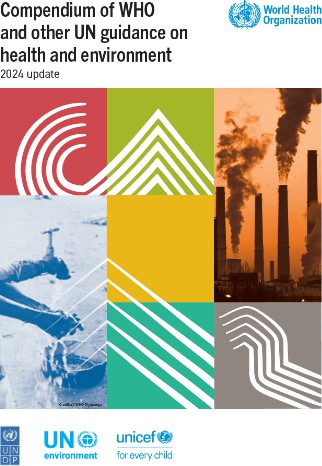 | | | This is a comprehensive collection of available guidance for decision makers. It includes over 500 prioritized actions and interventions for healthier environments and is regularly updated. This resource can be used once the identification of priority issues is done to select actions which can be implemented to address these issues. |
| --- | --- | --- | --- |
| **Aide-memoires** **–** **Summary** **guidance** **for** **country** **actions**^4^ | | | Briefings on various topics of environment, climate change and health. These provide background information, key actions for health protection, and links to more in-depth guidance to support country-level action, monitoring measurable results. |
|  | 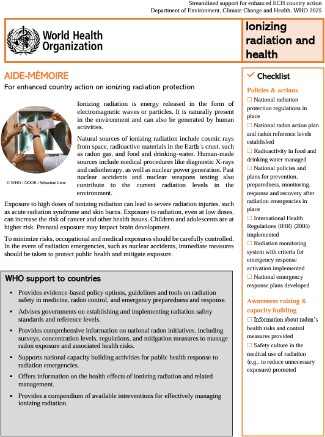 |  |  |
| **Safe,** **climate-resilient** **and** **environmentally**  **sustainable** **health** **care** **facilities:** **an** **overview**^5^ | | | This guide (i) provides an overview and definitions of safe, climate-resilient and  environmentally sustainable health care facilities; (ii) lists concrete guidance |

| 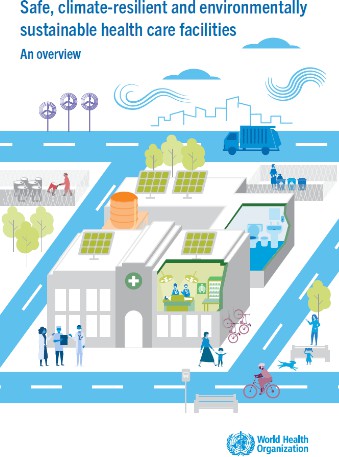 | about actions that can be taken to achieve them; and (iii) points to key materials, resources and tools that provide more detailed guidance and actions. This includes strengthening the health sector to position health in cross-sectoral policies and across environmental issues. It also involves making the sector increasingly resilient and prepared for the environmental crisis, while leading by example and reducing its own significant environmental impact. |
| --- | --- |
| **Training** **–** **Environment,** **climate** **change** **and** **health** **for** **practitioners** **and** **actors** **guiding** **policy** **change**^6^  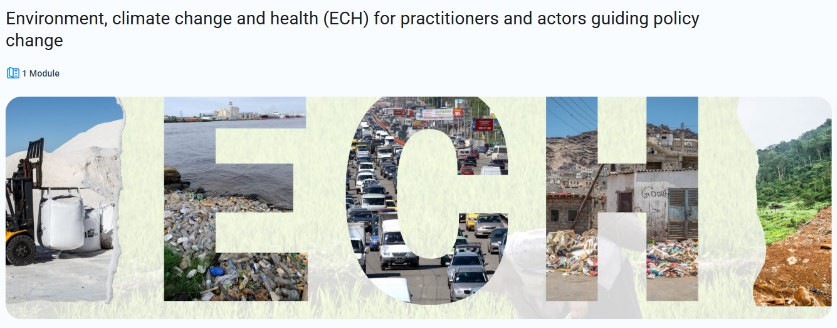 | Online, basic training on environment, climate change and health covering key facts, figures and available interventions on the most important environmental risks including climate change, air pollution, water, sanitation and hygiene, chemicals and others. |

**References**

1. Health and Environment Country Scorecards. Accessed May 26, 2025. https://[www.who.int/teams/environment-climate-change-and-](http://www.who.int/teams/environment-climate-change-and-) health/monitoring/scorecards
2. Checklist for rapid assessment of policies and actions in environment, climate change and health: scaling up country actions for environment, climate change and health. Accessed May 26, 2025. https://[www.who.int/publications/i/item/WHO-HEP-ECH-EHD-2023.1](http://www.who.int/publications/i/item/WHO-HEP-ECH-EHD-2023.1)
3. Compendium of WHO and other UN guidance on health and environment. Accessed May 26, 2025. https://[www.who.int/tools/compendium-](http://www.who.int/tools/compendium-) on-health-and-environment
4. Aide-memoires - briefs for country action. Accessed May 26, 2025. https://[www.who.int/teams/environment-climate-change-and-](http://www.who.int/teams/environment-climate-change-and-) health/interventions/aide-memoires---briefs-for-country-action
5. World Health Organization. *Safe,* *Climate-Resilient* *and* *Environmentally* *Sustainable* *Health* *Care* *Facilities:* *An* *Overview*. World Health Organization; 2024. Accessed May 23, 2025. https://iris.who.int/handle/10665/379483
6. WHO Academy. Environment, climate change and health (ECH) for practitioners and actors guiding policy change. Learning Experience Platform. 2025. Accessed May 26, 2025. https://whoacademy.org
